# Phosphodiesterase-4D Knock-down in the Prefrontal Cortex Alleviates Chronic Unpredictable Stress-Induced Depressive-Like Behaviors and Memory Deficits in Mice

**DOI:** 10.1038/srep11332

**Published:** 2015-07-10

**Authors:** Zhen-Zhen Wang, Wei-Xing Yang, Yi Zhang, Nan Zhao, You-Zhi Zhang, Yan-Qin Liu, Ying Xu, Steven P. Wilson, James M. O'Donnell, Han-Ting Zhang, Yun-Feng Li

**Affiliations:** 1 Department of New Drug Evaluation, Beijing Institute of Pharmacology and Toxicology, Beijing 100850, China; 2 State Key Laboratory of Bioactive Substances and Functions of Natural Medicines, Institute of Materia Medica & Neuroscience Center, Chinese Academy of Medical Sciences and Peking Union Medical College, Beijing 100050, China; 3 Department of Pharmacology, China Pharmaceutical University, Nanjing 210009, China; 4 Department of Anatomy, School of Preclinical Medicine, Beijing University of Chinese Medicine, Beijing 100029, China; 5 Department of Pharmacology, Physiology, and Neuroscience, University of South Carolina School of Medicine, Columbia, SC 29208, USA; 6 School of Pharmacy & Pharmaceutical Sciences, The State University of New York at Buffalo, New York, NY 14260, USA; 7 Departments of Behavioral Medicine & Psychiatry and Physiology & Pharmacology, West Virginia University Health Sciences Center, Morgantown, WV26506, USA

## Abstract

Phosphodiesterase 4 (PDE4) has four isoforms (PDE4A-D) with at least 25 splice variants. PDE4 subtype nonselective inhibitors produce potent antidepressant-like and cognition-enhancing effects via increased intracellular cyclic AMP (cAMP) signaling in the brain. Our previous data have demonstrated that long-form PDE4Ds appear to be involved in these pharmacological properties of PDE4 inhibitors in the normal animals. However, it is not clear whether long-form PDE4Ds are critical for the behaviors and related cellular signaling/neuronal plasticity/neuroendocrine alterations in the depressed animals. In the present study, animals exposed to the chronic unpredictable stress (CUS), a rodent model of depression, exhibited elevated corticosterone, depressive-like behavior, memory deficits, accompanied with decreased cAMP-PKA-CREB and cAMP-ERK1/2-CREB signaling and neuroplasticity. These alterations induced by CUS were reversed by RNA interference (RNAi)-mediated prefrontal cortex long-form PDE4Ds (especially PDE4D4 and PDE4D5) knock-down, similar to the effects of the PDE4 subtype nonselective inhibitor rolipram. Furthermore, these effects of RNAi were not enhanced by rolipram. These data indicate a predominant role of long-form PDE4Ds in the pharmacotherapies of PDE4 inhibitors for depression and concomitant memory deficits. Long-form PDE4Ds, especially PDE4D4 and PDE4D5, appear to be the promising targets for the development of antidepressants with high therapeutic indices.

Long-term exposure to unpredictable life stressors is a major precipitating factor in the development of depression in humans^1^. In addition, depression is generally accompanied by memory deficits which are critical determinants of functional outcome in this population^2^. Chronic unpredictable stress (CUS) is currently recognized as a valid model of depression and is typically used in rodents to resemble the human depressive-like state^3^ and concomitant memory deficits^4^.

Phosphodiesterase-4 (PDE4) inhibitors such as rolipram produce antidepressant-like^5^,^6^ and cognition-enhancing effects^7^,^8^,^9^,^10^ via cAMP signaling^6^,^11^. The cAMP signaling cascade is important in the mediation of neuroplasticity^12^, which is the neurochemical substrate of antidepressant efficacy^13^ and cognitive functions^14^. Although rolipram produces preclinical and clinical antidepressant efficacy, its therapeutic utility is limited by problematic side effects such as severe nausea and emesis^15^.

The PDE4 family is encoded by four genes (PDE4A-D) and multiple variants^16^,^17^. The PDE4D isoform has attracted considerable attention given that it is potential therapeutic target in the treatment of depression^18^,^19^,^20^ and memory deficits^11^,^21^,^22^,^23^. Unfortunately, deficiency of PDE4D also causes the emetic-like response^11^,^24^. This is consistent with the enrichment of PDE4D in the area postrema and nucleus tractus solitarius^25^, two structures that are known to be involved in the emetic response^26^. Excitingly, selective PDE4D allosteric modulators^22^ and PDE4D selective inhibitor^21^ incompletely inhibit PDE4 activity and enhance memory but have reduced potential to cause emesis.

We have previously indicated long-form PDE4D variants, especially PDE4D4 and PDE4D5, may play important roles in the mediation of antidepressant-like and cognition-enhancing effects but appear not to cause emesis in normal mice^11^,^20^. However, there is no evidence of long-form PDE4Ds on the alterations in neuroendocrine, impaired neuronal plasticity, cellular and behavioral disturbances in response to CUS. The gap between this promising pharmacotherapeutic target and the pathophysiology of depression hampers the development of PDE4D variant-selective inhibitors into novel antidepressants.

Hence, the present study was designed to investigate the roles of long-form PDE4Ds in antidepressant-like and cognition-enhancing effects employing the CUS model in mice. The RNA interference (RNAi) technique was applied to the long-form PDE4Ds knock-down. We hypothesized that long-form PDE4Ds played a predominant role in the pharmacotherapies of PDE4 inhibitors for depression and concomitant memory deficits.

## Results

### Effects of 4DmiR microinfusion on CUS-induced changes in expression of long-form PDE4D variants

**Figure 1:**
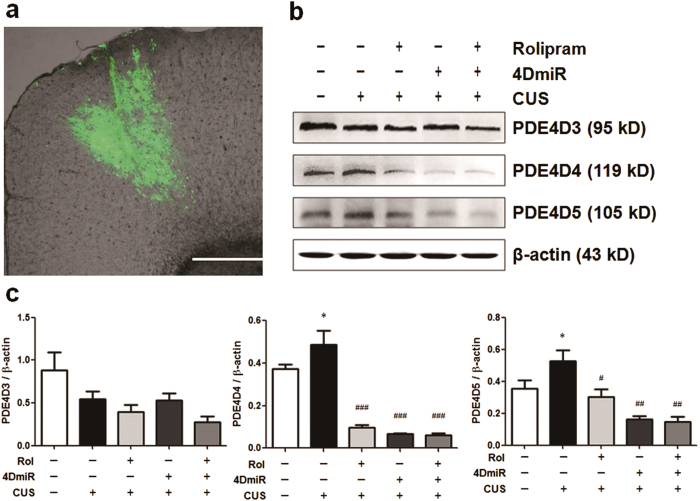
Effects of 4DmiR on CUS-induced changes in the expression of long-form PDE4D variants. (***a***) Microinjection sites and high, specific expressions of EGFP (green) in the prefrontal cortices observed under fluorescence microscopy. Scale bars = 500 μm. (***b***) Representative cropped immunoblots of long-form PDE4D variants (full-length blots are presented in Supplementary Figure S1). The prefrontal cortical tissues of 3 mm in diameter around the injection site were punched out for western blot analysis. All gels were run under the same experimental conditions and blots were processed in parallel. The boundary between the gels was delineated by a black line. (***c***) The histogram represents semi-quantitative results of western blot analysis. The values of densitometric analysis were normalized by the level of *β*-actin. Values shown are means ± SEM; n = 3-4; *P < 0.05, compared with non-stressed control (NC + Veh); ^#^*P* < 0.05, ^##^*P* < 0.01, ^###^*P* < 0.001 compared with CUS (NC + Veh).

The lentivirus-mediated microinfusion *in vivo* was traced by the high and specific expression of EGFP (Fig. 1a). The expressions of PDE4D4 and PDE4D5 in chronically stressed mice were significantly increased [F(4,14) = 18.48, *P* < 0.05 and F(4,14) = 6.49, *P* < 0.05, respectively], compared with the control. 4DmiR treatment reversed these increases and significantly down-regulated the levels of PDE4D4 (*P* < 0.001) and PDE4D5 (*P* < 0.01), similar to the effects of rolipram administration (*P* < 0.001 for PDE4D4 and *P* < 0.05 for PDE4D5). In addition, rolipram did not alter the effects of 4DmiR on the expressions of PDE4D4 and PDE4D5. The expression of PDE4D3 was not substantially changed by CUS exposure and 4DmiR treatment (Fig. 1b,c).

### Effects of 4DmiR on CUS-induced changes in neuroendocrine and physical state

**Figure 2:**
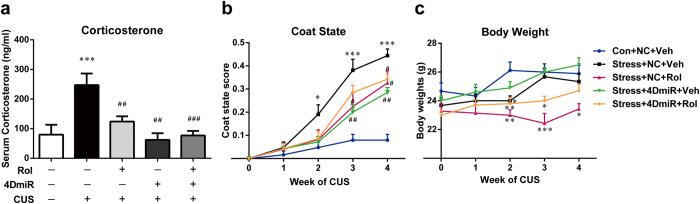
Effects of 4DmiR on CUS-induced changes in neuroendocrine and physical state. (***a***) CUS mice showed a significant increase of serum corticosterone concentration and this effect was reversed by 4DmiR and/or rolipram treatment. Serum corticosterone concentration was detected using a commercial enzyme-linked immunosorbent assay (ELISA) kit. (***b***) The evaluation of the coat state during each week of CUS revealed a significant degradation of the coat state, from week 2 to week 4, in stressed mice in comparison to non-stressed mice. Rolipram and/or 4DmiR treatment significantly reversed this effect. (***c***) There was no statistically difference in the body weight, except for week 2. While rolipram alone or in combination with 4DmiR significantly disrupted the normal gain in body weight as compared with the control starting from week 2. 4DmiR alone did not affect the body weight gain. Values shown are means ± SEM; n = 7–10; *P < 0.05, **P < 0.01, ***P < 0.001 compared with non-stressed control (NC + Veh); ^#^*P* <* *0.05, ^##^*P* < 0.01, ^###^*P* < 0.001 compared with CUS (NC + Veh).

Following the CUS regimen, the serum corticosterone level in chronically stressed mice was higher than that in non-stressed mice [Fig. 2a; F(4, 22) = 8.005, *P* < 0.001]. 4DmiR treatment attenuated this alteration and significantly reduced corticosterone concentrations, compared with vehicle/NC-treated CUS mice (*P* < 0.01), similar to the effect of rolipram administration (*P* < 0.01). In addition, the effect of 4DmiR on the serum corticosterone level was not changed in the presence of rolipram.

CUS led to a significant deterioration of coat state from week 2 to week 4 as demonstrated by an increased coat state score [Fig. 2b; week 2: F(4, 40) = 2.893, *P* < 0.05; week 3: F(4, 40) = 8.344, *P* < 0.001; week 4: F(4, 40) = 21.33, *P* < 0.001]. 4DmiR (*P* < 0.01) or rolipram (*P* < 0.05) significantly prevented this degradation from the third week. And the effect of 4DmiR plus rolipram on coat state was observed at the fourth week (*P* < 0.05).

Body weight was measured before the onset of the CUS regimen and then weekly until the end of the CUS procedure. CUS mice showed a reduction of the body weight gain only at the second week [Fig. 2c; F(4, 40) = 5.063, *P* < 0.05)]. While rolipram alone or in combination with 4DmiR significantly disrupted the normal gain in body weight as compared with the control starting from the second week [week 2: F(4, 40) = 5.063, *P* < 0.01 for rolipram alone or in combination with 4DmiR; week 3: F(4, 40) = 7.985, *P* < 0.001 for rolipram alone, *P* < 0.05 for rolipram plus 4DmiR; week 4: F(4, 40) = 4.892, *P* < 0.05 for rolipram alone]. 4DmiR alone did not affect the body weight gain.

### Effects of 4DmiR on CUS-induced depressive-like behaviors

**Figure 3:**
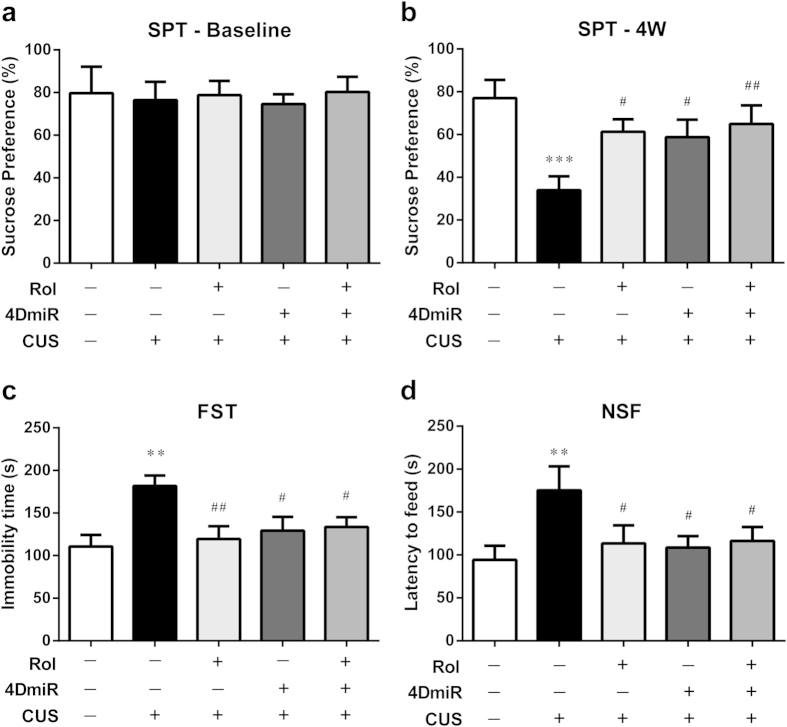
Effects of 4DmiR on CUS*-*induced changes in antidepressant-like behaviors. (***a***, ***b***) CUS paradigm substantially decreased the sucrose preference of mice and this alteration was reversed by 4DmiR and/or rolipram treatment. (***c***,***d***) Chronically stressed mice displayed significantly increases of the immobility time in the FST and the latency to feed in the NSF test compared with non-stressed mice. Rolipram and/or 4DmiR treatment significantly reversed these effects. Values shown are means ± SEM; n = 7–10; **P < 0.01 compared with non-stressed control (NC + Veh); ^#^*P* < 0.05, ^##^*P* < 0.01 compared with CUS (NC + Veh).

Anhedonia which is defined by decreased sucrose preference is the core symptom of depression and is suitable for monitor the depressive-like state in rodents^27^. To define the dynamics of the CUS response, the sucrose preference of each mouse was evaluated weekly. Initially, all groups of mice had a similar sucrose preference in baseline conditions (before stress, Fig. 3a). While a significant drop of sucrose preference was observed after 4-week stress procedure [Fig. 3b; F(4, 40) = 4.080, *P* < 0.001]. 4DmiR treatment reversed this alteration and significantly increased the sucrose preference compared with vehicle/NC-treated CUS mice (*P* < 0.05), similar to the effect of rolipram administration (*P* < 0.05). In addition, the effect of 4DmiR on the sucrose preference was not changed in the presence of rolipram.

Assessing the behaviors of mice, we found a 64.1% increase of the immobility time in the FST [Fig. 3c; F(4, 40) = 3.880, *P* < 0.01] and a 85.9% increase of the latency to feed in the NSF test [Fig. 3d; F(4, 40) = 2.839, *P* < 0.01] after the CUS procedure. 4DmiR treatment attenuated these alterations and significantly reduced the immobility time in the FST (*P* < 0.05) and the latency to feed in the NSF test (*P* < 0.05). A similar tendency was observed in mice treated with rolipram (FST: *P* < 0.01; NSF: *P* < 0.05). In addition, the effects of 4DmiR on the immobility time and the latency to feed were not changed in the presence of rolipram.

### Effects of 4DmiR on CUS-induced memory deficits

**Figure 4:**
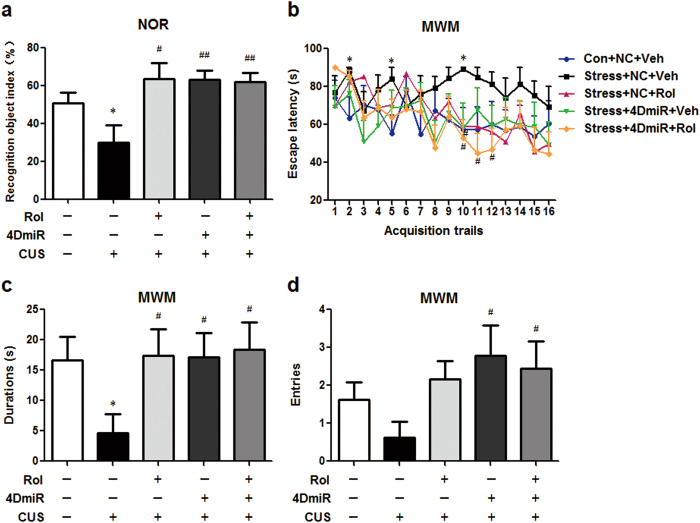
Effects of 4DmiR on CUS-induced memory deficits. (***a***) Memory deficits were observed in the NOR test in mice submitted to the CUS procedure. The decreased recognition index in the CUS group was reverted by 4DmiR and/or rolipram treatment. (***b***) Escape latency during the acquisition trials (6 trials × 2 days plus 4 trials × 1 day) in the MWM task in mice. (***c***, ***d***) Chronically stressed mice displayed significantly decrease in durations but not entries in the target quadrant in the probe trial of the MWM task. Rolipram and/or 4DmiR treatment significantly reversed these effects. Values shown are means ± SEM; n = 7–10; *P < 0.05 compared with non-stressed control (NC + Veh); ^#^*P* < 0.05, ^##^*P* < 0.01 compared with CUS (NC + Veh).

In the NOR test, mice exposed to stressors showed impairment in recognition memory revealed by a significant decrease in the recognition index in comparison with the controls [Fig. 4a; F(4, 40) = 5.502, *P* < 0.05] and this effect was reversed by 4DmiR treatment (*P* < 0.01). Similar result was also observed in mice treated with rolipram (*P* < 0.05) and the effect of 4DmiR on the recognition index was not changed in the presence of rolipram.

In the MWM task, during the 3-day acquisition training, all the mice displayed progressive decreases in the latency to find the submerged platform over training trials. Mice exposed to CUS had longer latencies to reach the platform than the controls [Fig. 4b; Trial 2: F(4, 40) = 2.147, *P* < 0.05; Trial 5: F(4, 40) = 1.303, *P* < 0.05; Trial 10: F(4, 40) = 1.887, *P* < 0.05]. And the stressed mice treated with 4DmiR alone or plus rolipram spent less time to find the submerged platform [Trial 10: F(4, 40) = 1.887, *P* < 0.05; Trial 11: F(4, 40) = 1.897, *P* < 0.05; Trial 12: F(4, 40) = 1.281, *P* < 0.05]. In the probe trial test performed 24 h after the last acquisition trial, stressed mice displayed robust decreases in durations [F(4, 40) = 2.595; *P* < 0.05] but not entries [F(4, 40) = 2.347; *P* > 0.05] in the target quadrant, as shown in Fig. 4c,d. 4DmiR treatment reversed this alteration and significantly increased the durations and entries in the target quadrant compared with vehicle/NC-treated CUS mice (*P* < 0.05 for both), similar to the effect of rolipram administration (Durations: *P* < 0.05; Entries: *P* > 0.05). In addition, the effects of 4DmiR on the durations and entries were not changed in the presence of rolipram.

### Effects of 4DmiR on CUS-induced deficits in dendritic complexity and spine density

**Figure 5:**
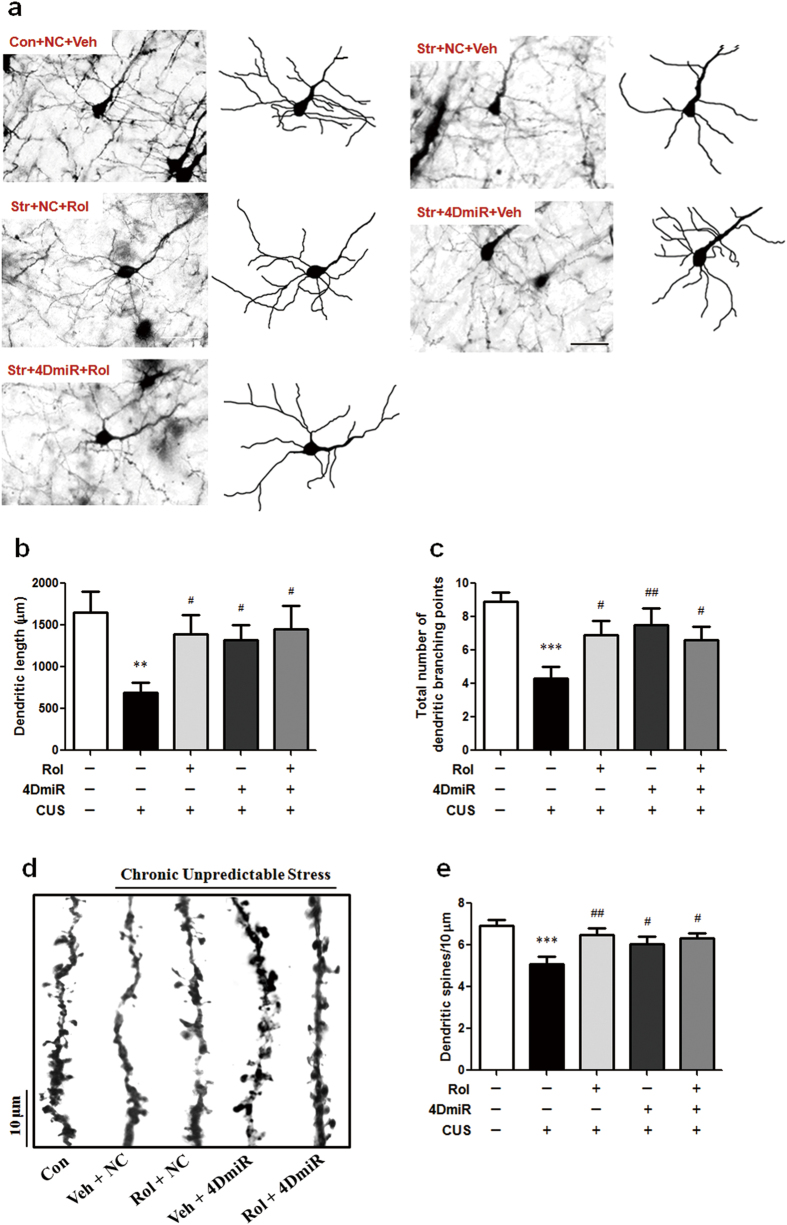
Effects of 4DmiR on CUS-induced changes in the dendritic complexity and spine density. (***a***) Representative photomicrograph of Golgi-impregnated cortical pyramidal neurons in the region of infection from animals of each group. Scale bar = 50 μm. (***b***,***c***) Summary data showed that the decreases in the total dendritic length and the number of branching points induced by CUS were reversed by 4DmiR treatment. These effects were similar to those of rolipram administration and were not significantly enhanced in the presence of rolipram. (***d***) Examples of dendrite fragments with visible spines from different groups. Scale bar = 10 μm. (***e***) The reduction of spine density induced by CUS was rescued by 4DmiR treatment, similar to the effect of rolipram administration. And this effect was not significantly enhanc***e***d in the presence of rolipram. Values shown are means ± SEM; n = 4; **P < 0.01, ***P < 0.001 compared with non-stressed control (NC + Veh); ^#^*P* < 0.05, ^##^*P* < 0.01 compared with CUS (NC + Veh).

The Golgi staining in Fig. 5 showed the significant decreases of total dendritic length [F(4,15) = 4.591; *P* < 0.01], branching points [F(4,15) = 6.187; *P* < 0.001] and spine density [F(4,15) = 5.550; *P* < 0.001] in mice subjected to the CUS procedure, compared with the control group. The dendritic complexity and spine density were dramatically affected and showed a significant increase in 4DmiR treated mice (*P* < 0.01 for branching points; *P* < 0.05 for dendritic length and spine density), compared with the CUS group. These effects were similar to those of rolipram administration (*P* < 0.01 for spine density; *P* < 0.05 for dendritic length and branching points) and were not considerably enhanced in the presence of rolipram. These results indicate that long-form PDE4Ds are important in the effects of rolipram reversing CUS-induced decreases of dendritic complexity and spine density.

### Effects of 4DmiR on CUS-induced changes in cAMP signaling

**Figure 6:**
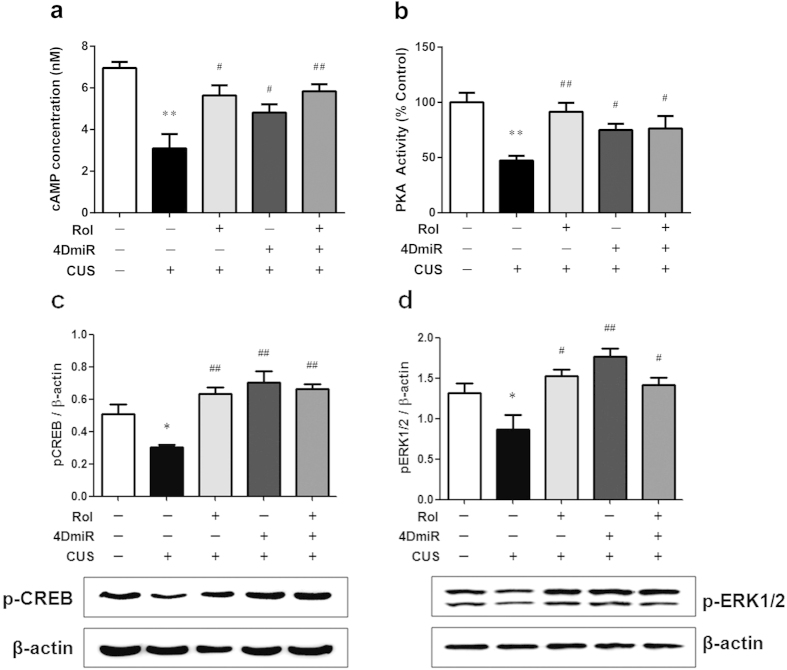
Effects of 4DmiR on CUS-induced changes in CREB transduction cascade. The prefrontal cortical tissues of 3 mm in diameter around the injection site were dissected for cAMP, PKA and western blot assays. (***a***, ***b***) CUS significantly decreased the cAMP concentration and PKA activity in the prefrontal cortices, whereas 4DmiR and/or rolipram treatment significantly reversed this effect. (***c***, ***d***) Western blot analysis demonstrated that CUS significantly decreased the protein levels of pCREB and pERK when no significant difference was revealed between treatments in the total CREB and ERK levels. Rrolipram and/or 4DmiR treatment significantly reversed CUS-induced cAMP-PKA-CREB and p44/42MAPK- CREB signaling dysfunction. Lower panels are representative cropped immunoblots detected by western blot (full-length blots are presented in Supplementary Figure S2) and upper panels are the corresponding quantifications. All gels were run under the same experimental conditions and blots were processed in parallel. The boundary between the gels was delineated by a black line. The values of densitometric analysis were normalized by the level of *β*-actin. Values shown are means ± SEM; n = 3-4; *P < 0.05, **P < 0.01 compared with non-stressed control (NC + Veh); ^#^*P* < 0.05, ^##^*P* < 0.01 compared with CUS (NC + Veh).

As shown in Fig. 6, cAMP concentration, PKA activity, and the levels of pCREB and pERK in the PFC were significantly decreased by CUS procedure [Fig. 6a–d; F_cAMP_ (4, 14) = 9.045, *P* < 0.01; F_PKA_(4, 14) = 5.355, *P* < 0.01; F _pCREB_ (4, 14) = 12.32, *P* < 0.05; F _pERK_ (4, 14) = 8.53, *P* < 0.05], whereas 4DmiR treatment reversed these alterations and significantly increased cAMP concentration (*P* < 0.05), PKA activity (*P* < 0.05), and the levels of pCREB and pERK (*P* < 0.01 for both), similar to the effects of rolipram administration (*P* < 0.01 for pCREB and PKA; *P* < 0.05 for cAMP and pERK). In addition, the effects of 4DmiR on the regulation of cAMP-PKA-CREB and cAMP-ERK1/2-CREB signaling were not substantially changed in the presence of rolipram. These results suggest that long-form PDE4Ds are important in the effects of rolipram reversing CUS-induced cAMP-PKA-CREB and cAMP-ERK1/2-CREB signaling dysfunction.

### Discussion

In the present study, the CUS model of mice successfully copied the depressive-like state by reducing the sucrose preference and worsening the coat state, accompanying with the elevation of the serum corticorsterone concentration. The animals exposed to the CUS exhibited behavioral deficits in tests measuring depression and memory. Furthermore, dendritic retraction and loss of spines, as well as down-regulation of cAMP, PKA, pCREB and pERK in the PFC were simultaneously observed in the CUS mice. Lentiviral vector-delivered shRNA caused the selective down-regulation of long-form PDE4D4 and PDE4D5 in the PFC, consistant with previous studies^11^,^20^,^23^. While the other long-form PDE4Ds (*i.e.* PDE4D7, 9) were not examined because of the lack of specific antibodies, but their contributions cannot be excluded. The behavioral, cellular and molecular alterations induced by the CUS were attenuated or reversed by the long-form PDE4Ds knock-down in the PFC, similar to the PDE4 inhibition by rolipram. Moreover, these effects of long-form PDE4Ds knock-down were not affected by chronic rolipram treatment. We have previously indicated that locomotor activity of mice was not affected by long-form PDE4Ds knock-down in the hippocampus and PFC^11^,^20^, suggesting that the observed behavioral differences were not due to potential locomotor activity changes. Together, our data support the hypothesis that long-form PDE4Ds, especially PDE4D4 and PDE4D5, are the pivotal variants responsible for reverse effects of PDE4 inhibition in the depressive-like symptoms and concomitant memory deficits employing the CUS model.

The CUS paradigm is an animal model of depression with good predictive validity (behavioral alterations are reversed by chronic treatment with most current antidepressants), face validity (almost all the symptoms of depression are demonstrated), and construct validity (CUS causes anhedonia, the core symptom of depression)^28^. Anhedonia is defined as “the decreased capacity to experience pleasure of any sort”^29^. In an attempt to elucidate the effects of long-form PDE4Ds on the depressed animals, we adopted the CUS model in mice. This 4-week design offered a closer resemblance to the chronically stressful life events which are the major risk factor for developing depression^30^. The effectiveness of this procedure was monitored by the decrease in the responsiveness to rewards (sucrose preference). It was also confirmed by the elevated serum corticosterone, the degradation in the physical coat state, and behavioral deficits in the FST and the NSF. These alterations were consistent with previous studies^3^,^28^. BALB/c mice were used in this experiment for the reason that this strain is more vulnerable to stress across a wide range of behavioral tests^31^,^32^ and more easy to induce the memory deficits^33^ than other strains of mice after CUS exposure.

Although the sucrose preference was substantially different between the control and CUS groups, there existed no statistical difference in the body weights between the two groups at the end of CUS procedure in the present study. This result is in accordance with some previous studies^34^, although the weight loss is reported in the majority of published studies^35^. The explanations for this discrepancy may be due to the different CUS protocols, species/strains and time points in the different laboratories. Additionally, it has been demonstrated that CUS induces a generalized decrease in hedonic responses, whereas weight loss is variable across experiments^36^. Another important evidence supporting the independence of these two measures is that chronic antidepressant treatment normalizes the sucrose preference, but not the body weights^37^. Thus, as the validation of successful CUS model, the changes in the sucrose preference are more convincing than the changes in body weights. The elevation in serum corticorsterone levels, the deterioration of coat state and the behavioral impairments in the FST and the NSF further supported the successful experimental protocol of the CUS procedure.

The implications of depression and memory deficits have been revealed by sufficient evidence^38^. In this study, we investigated the effects of long-form PDE4Ds on the impairments of spatial working memory and object recognition memory in the mouse CUS model of depression. It was found that CUS exposure induced memory deficits in BALB/c mice, which is consistent with previous studies^33^. Stressed BALB/c mice showed a poor learning performance and impaired spatial memory in the MWM, as well as impaired object recognition memory in the NOR. Knock-down of long-form PDE4Ds in the PFC, a brain region important for mediating cognition associated with depressive disorders^39^, restored the CUS-induced memory deficits in the MWM and the NOR. Furthermore, these effects were not altered in the presence of rolipram, indicating a predominant role of long-form PDE4Ds in the reversal of memory deficits induced by CUS exposure.

It is well established that loss of neuronal plasticity is involved in the pathophysiology of depression and memory deficits^14^. This study illustrated that CUS exposure contributed to the dendritic retraction, simplicity and spine loss in the PFC. The morphological changes were also associated with depressive-like behaviors, as well as the impaired spatial memory and object recognition memory. Our findings are in accordance with previous studies^14^,^40^. We also demonstrated that the disrupted neuronal plasticity in the PFC were reversed or prevented by the long-form PDE4Ds knock-down. This effect was not further enhanced by the inhibition of the remained PDE4 subtypes and variants by rolipram, supporting the important role of long-form PDE4Ds in the counteraction of stress-induced neuronal plasticity disruption.

CREB is activated by the phosphorylation of its serine 133 (Ser133) region, which is the binding site of multiple kinases including cAMP-dependent PKA and mitogen-activated protein kinase (MAPK)^41^. It is noted that cAMP-PKA signaling pathway is one of the major signaling transduction mechanisms that participate in depression^42^ and memory deficits^43^. The ERK1/2 signaling pathway is proposed to be the best studied MAPK signaling cascade in depression^42^. We found that cAMP, PKA, pERK and pCREB were all marked reduced response to the CUS procedure in the PFC, which are supported by previous studies^44^,^45^. The attenuated cAMP-PKA-CREB and cAMP-ERK1/2-CREB signaling pathways underlie stress-induced behavioral deficits and neuronal plasticity disruption^44^, although the contradictory effects have also been observed^46^. However, some CUS models fail to alter ERK1/2 signaling or lead to the hyperphosphorylation of ERK1/2 in the PFC^47^,^48^. Another contrary finding to those above exist indicates that sustained elevation of cAMP signaling causes spine loss in the PFC^49^. The discrepancy may be due to the diversity and complexity of the roles of signaling molecules in stress, as well as the type of stress and its duration. The different durations of stress exposure lead to the proper response or the adaptive response to CUS paradigm. Here we further demonstrated the reversed expression of these CREB signaling cascade members after stress exposure by the long-form PDE4Ds knock-down. These effects were not enhanced in the presence of rolipram, indicating the crucial role of long-form PDE4Ds in the restoration of down-regulated cAMP-PKA-CREB and cAMP-ERK1/2-CREB signaling pathways. Long-form PDE4Ds contain an ERK phosphorylation site in the catalytic domain and a PKA phosphorylation site in the upstream conserved regions 1 (UCR1), which is distinguished from other variants^6^. ERK phosphorylation transiently inhibits activity of long-form PDE4Ds^50^,^51^, followed by a rise in cAMP. The increased cAMP causes PKA to phosphorylate UCR1, thereby overcoming its inhibition by pERK^51^,^52^. ERK phosphorylation acts on PDE4 to regulate cAMP signaling either negatively or positively depending on the expression pattern and localization of long-form PDE4s^53^. Thus, long-form PDE4Ds maybe mediate the cross-talk between the cAMP-PKA-CREB and cAMP-ERK1/2-CREB signalling pathways, which is supported by the findings that PDE4 enzymes orchestrate various signaling cross-talk^53^.

Taken together, this study provides the first experimental evidence of (i) the alteration of long-form PDE4Ds in the PFC of mice subjected to CUS, (ii) the dominant roles of long-form PDE4Ds, especially PDE4D4 and PDE4D5, in the reversal of CUS-induced depressive-like behaviors, memory deficits, neuronal plasticity disruption and hypofunction of cAMP-CREB signaling. Thus, our findings could benefit the structure- and scaffold-based design of PDE4D variant-selective inhibitors with high therapeutic indices for treatment of depression and disorders with memory deficits.

## Methods

### Animals

Adult male BALB/c mice initially weighing 20–24 g (Vital River Laboratories, Beijing, China) were maintained under a 12-h light/ dark cycle at room controlled temperature (22 ± 2 °C) with free access to food and water except as mentioned below. All mice were group-housed for 1 week prior to the beginning of the experiment and handled daily throughout the experiment to minimize the effects of non-specific stress. All experiments were performed in accordance with the National Institute of Health Guide for the Care and Use of Laboratory Animals (NIH Publications No. 80–23) and were approved by the Animal Care and Use Committees of Beijing Institute of Pharmacology and Toxicology. Efforts were made to minimize suffering and to reduce the number of animals used.

### Materials

Rolipram was purchased from Sigma-Aldrich (St. Louis, MO, USA). Lentiviral vectors containing the non-targeting negative control (NC) sequence or shRNA sequence in a miRNA scaffold targeting long-form PDE4D (4DmiR) were designed using Invitrogen BLOCK-iT Pol II miR RNAi expression vector kits and the associated software. The sequence for the PDE4D miRNA (shRNA-mir hairpin structure) was 5’-AATGGAGTCACAATCAAGTCAGTTTTGGC-

CACTGACTGACTGACTTGAGTGACTCCATT-3’. The NC sequence from Invitrogen was 5’-GAA ATGTACTGCGCGTGGAGACGTTTTGGCCACTGACTGACGTCTCCACGCAGTACATTT-3’. Invitrogen NC sequences were used as the control of PDE4D miRNAs. All the miRNAs and NC sequences were cloned into lentiviral transfer vectors and driven by the phosphoglycerate kinase-1 promoter. The vectors contained enhanced green fluorescence protein (EGFP) as a reporter for tracking lentivirus-mediated expression as described previously^11^.

### Experimental design

**Figure 7:**
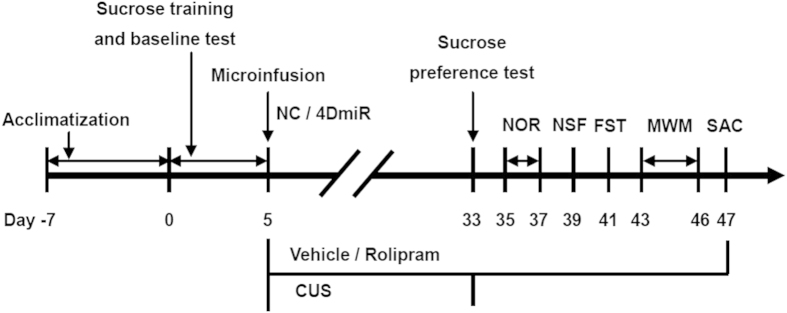
Experimental design. Mice were provided 5 days to establish a stable baseline of sucrose consumption following the 1-week acclimatization. After lentiviral vectors harbouring the NC or 4DmiR sequence were microinfused into bilateral prefrontal cortices, mice were housed in normal conditions (control) or subjected to the chronic unpredictable stress (CUS) procedure for 28 days. Behavioural experiments including the sucrose preference test (SPT), the novel object recognition (NOR) test, the novelty suppressed feeding (NSF) test, the forced-swim test (FST) and the Morris water-maze (MWM) test were performed after the CUS procedure. Rolipram (1.25 mg/kg) or its vehicle (saline containing 2.5% DMSO) was injected (i.p.) once daily, beginning from 6 h after the viral infusions (day 5) and continuing until d47 when the animals were sacrificed (SAC) for biochemical assays.

A total fifty mice were randomly distributed into five groups (n = 10): (i) NC + vehicle (Veh; 2.5% DMSO) + control (CON); (ii) NC + Veh + CUS; (iii) NC + rolipram (Rol, 1.25 mg·kg−1) + CUS; (iv) 4DmiR + Veh + CUS; and (v) 4DmiR + Rol + CUS. The dose of rolipram was chosen based on our previous studies^5^,^11^. After 1 week of acclimatization, mice were provided 5 days to establish a stable baseline of sucrose consumption. Then NC or 4DmiR was microinfused into bilateral prefrontal cortices of mice. One group was housed in normal conditions (control) and the other groups were subjected to CUS. After the 4-week CUS procedure, behavioral performances including the sucrose preference test (SPT), the novel object recognition (NOR) test, the novelty suppressed feeding (NSF) test, the forced-swim test (FST) and the Morris water-maze (MWM) task were assessed on different days. Rolipram or vehicle was administered (i.p.) once a day from the second week and continued up to the end, as illustrated in Fig. 7. Given the sedative effects of rolipram at 1.25 mg·kg^−1^ are absent 1 h after administration^5^,^11^, training or testing was conducted 1 h after rolipram treatment. After behavioral tests, 3–4 animals per group were used for biochemical analysis, 4 animals per group for Golgi staining and 1–2 animals per group for the fluorescence observation of injection sites.

### Mouse surgery and lentiviral microinfusions

Mice were weighed and anesthetized with ketamine (100 mg/kg, i.p.) and xylazine (20 mg/kg, i.p.) before placed in a stereotaxic apparatus (Stoelting, Wood Dale, IL, USA). All the surgical supplies were sterilized before the operation. The bilateral prefrontal cortices infusions were performed via a 10 μl Hamilton microsyringe with a 30-gauge needle fitted to the arm of the stereotaxic apparatus. An incision was made in the scalp and a hole was drilled in the skull over the injection site, following the coordinates^54^: the bilateral prefrontal cortices: anterior–posterior (AP, from Bregma) + 1.5 mm, medial–lateral (ML, from midline) ± 0.5 mm, and dorsal–ventral (DV, from dura) −1.2 mm from dura. The 30-gauge-needle was lowered into the dorsal limb of the prefrontal cortex (PFC). Lentiviral vectors containing NC or 4DmiR (4 × 10^6^ TU/μl, 1 μl/side) were infused at a rate of 0.2 μl/min using a UMP3 microsyringe injector and Micro4 controller (World Precision Instruments, Sarasota, FL, USA). The needle was slowly retracted after additional 5 min in order to assure adequate diffusion of the vectors.

### Chronic unpredictable stress procedure

Chronic unpredictable stress regimen was modified from that previously described in mice^55^. Mice were subjected to different kinds of stressors for four consecutive weeks: (1) cold swimming (10 °C); (2) cage tilting (45°); (3) restraint stress; (4) overhanging; (5) foot shock (0.8 mA, 5-s duration, 60-s inter-shock interval); (6) white noise (110 dB); (7) tail pinch (1 cm apart from the end of the tail); (8) damp sawdust (200 ml water absorbed in sawdust bedding); (9) sawdust-free cage; (10) sawdust-free cage with 200 ml water (21 °C); (11) continuous cage shaking (high speed horizontal shaking); (12) overnight illumination and (13) water or food deprivation. Two or three stressors were applied daily: once in the morning (beginning at 0900 hours), once in the afternoon (beginning at 1400 hours), and overnight. The stress procedure in the first two weeks was presented in Table 1 and repeated in an unpredicted manner during the following two weeks. Control mice were housed in a separate room, having no contact with the stressed mice.

### Serum corticosterone levels

Serum corticosterone concentration was determined using commercially available corticosterone enzyme immnuoassay kits (Enzo Life Sciences, Farmingdale, NY, USA) according to the manufacturer's instructions. Following behavioral testing, mice were sacrificed by decapitation. Blood samples were collected and allowed to clot. Serum samples were separated by centrifugation (3000 rpm, 15 min) at 4 °C and stored at −20 °C until time of assay. Considering the circadian rhythm^56^, mice were bled between 10:00 and 12:00 h for measurement of corticosterone levels.

### Coat state and body weight

To define the dynamics of the CUS response, the coat state and the body weight of each animal were evaluated weekly^57^. The coat-state evaluation by observers unaware of treatments involved the assessment of five different body parts: dorsal coat, ventral coat, tail, forepaws and hind paws (head and neck were not included for the reason of surgery). For each body part, a score of 0 was given for a well-groomed coat and 1 for an unkempt (dirty, piloerection, greasy, fluffy or less dense) coat. The final score was obtained by adding the scores for each body part and dividing by the total number of body parts. The final score has been pharmacologically validated as an index of the general physical state of BALB/c mice.

### Behavioral procedures

#### Sucrose Preference Test (SPT)

Mice were subjected to the sucrose preference test before (baseline measurement) and after the application of chronic stress procedure. A 5-day sucrose preference protocol was conducted as previously described^58^,^59^. Briefly, mice were individually housed and habituated to two identical bottles filled with tap water (water/water) on days 1 and 2 or 1% sucrose solution (sucrose/sucrose) on days 3 and 4. On day 5, mice were given a free choice between two bottles for 10 h, one with 1% sucrose solution and another with tap water. To minimize the effect of side preference, the position of the bottles was switched in the middle of the test (5 h). To prevent the spillage of liquids resulting from temperature difference, bottles were filled in advance and the temperatures between the room and the drinking solutions were balanced. No food and water deprivation was applied before the test. Sucrose preference was calculated as sucrose consumed/(sucrose consumed + water consumed) × 100%.

#### Novelty-Suppressed Feeding (NSF) Test

The NSF test was performed during a 5-min period as described previously^5^. In brief, mice were deprived from food for 24 h and then were individually placed in a corner of the white plexiglas chamber (54 × 28 × 21 cm) for 5 min. The floor was covered with 2 cm of sawdust. Four pellets of food (regular chow) were placed in the center of the floor. The latency to eat (defined as the mouse chewing or biting the pellet with the forepaws, but not merely sniffing or toying with the pellet) the food was recorded.

#### Forced-Swim Test (FST)

The test was performed in mice as described previously^60^,^61^. Briefly, mice were gently introduced into a glass cylinders (20 cm diameter, 45 cm height) filled with water (23 ± 1 °C; depth 28 cm) for 6 min. Immobility time (the time during which mice floated motionlessly only with the small movements necessary to keep their heads above the water) was recorded during the last 4 min period of the test.

#### Novel Object Recognition (NOR) Test

The NOR test was carried out as previously described^11^. Experiments were conducted in a dimly lit room. During the habituation session, mice were allowed to become familiar with the testing box for 5 min. Twenty-four hours later, mice were again placed in the same apparatus and were individually exposed to two identical objects placed in two corners of the box for 5 min (the sample session). After another 24-h interval, mice were returned to the box with a previously presented familiar object and a novel one for 5 min (the test session). Exploration was defined as initiatively facing, sniffing or touching the object (within 2 cm from the object). The accumulative time exploring each object (Tf and Tn for familiar and novel objects, respectively) was recorded for determination of the recognition index [RI = Tn/(Tn + Tf)].

#### Morris Water-Maze (MWM) Task

The MWM task was performed following the procedures described in earlier studies^11^,^62^. Behavioral testing was conducted in a circular pool (100 cm diameter, 35 cm depth), which was divided into four equally spaced quadrants. The pool was filled to the depth of 17 cm with water (21 ± 1 °C) made opaque by addition of powdered milk. A circular platform was placed in the center of one of the quadrants 1.5 cm below the surface of the water. The pool was located in a well-lit room with some external cues, which remained in the same location throughout the acquisition and the probe trials. Mice were individually placed in the pool facing the wall at different starting points, except for the target quadrant containing the hidden platform. Animals were trained to escape by swimming and climbing onto the platform during the acquisition trials (6 trials ×  2 days plus 4 trials × 1 day; 16 trials in total). Those failed to find the hidden platform within 90 s were guided towards it and stayed there for 30 s before being removed. The latency to escape by swimming onto the platform was recorded. Twenty-four hours after the last acquisition training trial, the probe trial was conducted in the absence of the platform. The number of entries and the time spent in the target quadrant were recorded with the cut-off time 60 s.

### Golgi impregnation and dendrite analysis

FD Rapid GolgiStai kit (FD NeuroTechnologies, Ellicott City, MD, USA) was used for Golgi impregnation of tissue. Golgi staining was performed according to manufacturer’s instructions. In brief, blocks were impregnated into solutions A and B for 2 weeks at room temperature in the dark. Seventy-two hours after placing in solution C (4 °C, in the dark), coronal cryostat sections (100 μm) were cut from the PFC (±0.25 mm from the coronal section through the injection site in rostro-caudal direction, a total of four to five sections per animal) on a freezing microtome (Leica, Wetzlar, Germany) and mounted onto gelatinized slides. Following drying in the dark, sections were rehydrated, reacted in solutions D and E, and dehydrated with 50, 75, 95, and 100% ethanol, respectively. Finally, sections were cleared in xylene and coverslipped with resinous mounting media.

For quantitative analysis, five to six neurons from each animal and four animals per group were analyzed. The criteria used to select neurons for reconstruction have been fully described previously^63^. Briefly, neurons chosen for analysis had to be well impregnated, clearly distinguishable from adjacent cells or precipitant, located in the region of infection and have continuous unbroken dendrites. Neurons were digitally reconstructed, traced using Image-Pro Plus software, Version 7.0 (Media Cybernetics, Bethesda, MD, USA). The total dendritic length and the number of branching points were measured by Sholl analysis^64^. Spines were counted at high magnification (100 × oil objective). Only branches over 20 μm in length were included in the study. Spine density was calculated per 10 μm of dendritic length.

### Western-blot analysis

Western blotting was performed as described previously^65^ with minor modifications. Punched prefrontal cortical tissues (3 mm in diameter around the injection site on both sides) were extracted by RIPA lysis buffer (Millipore, Billerica, MA, USA) plus protease inhibitor and phosphatase inhibitor cocktail (Thermo Pierce, Rockford, IL, USA). Fifty microgram of total protein was resolved by electrophoresis, transferred onto nitrocellulose membranes and blocked by 5% skimmed milk solution. The membranes were incubated with rabbit antibody against PDE4D3, PDE4D4, PDE4D5 (all at a dilution of 1:500; FabGennix, Frisco, TX, USA), cAMP response element-binding (CREB) protein, pCREB (both at a dilution of 1:500; Millipore, Billerica, MA, USA) or β-actin (at a dilution of 1:3000; Santa Cruz Biotechnology, Santa Cruz, CA, USA) at 4 °C overnight. After washing and incubating with secondary antibodies (at a dilution of 1:3000; Santa Cruz Biotechnology, Santa Cruz, CA, USA), the specific bands were detected and quantified using Gel-Pro Analyzer software, Version 3.1 (Media Cybernetics, Bethesda, MD, USA).

### cAMP assay

The lance cAMP kit (PerkinElmer, Waltham, MA, USA) was used to determine intracellular cAMP concentrations of the lysed samples. The assays were performed following the manufacturer’s instruction in 384-well format. Counts at 665 nm obtained in cAMP standard curves allowed the quantitative determination of the cAMP concentration in samples. The signal at 615 nm is useful to identify dispensing or quenching problems. All experiments were performed in the presence of 0.05% bovine serum albumin and 0.5 mM isobutyl-methyl-xanthine to allow accumulation of cAMP.

### Protein kinase A (PKA) activity assay

PKA activity was determined using a non-radioactive protein kinase assay kit (Enzo Life Sciences, Farmingdale, NY, USA) according to the manufacturer’s recommendations. Lysed samples were added to the appropriate wells according to ELISA protocol and the purified active PKA included with the kit was used as the positive control. The assay was developed with tetramethylbenzidine substrate and the color development was stopped with acid stop solution. The intensity of the color was measured at 450 nm in a microplate reader. The results were presented as the percentage of PKA activity in each group to that in the control group.

### Statistical analysis

The data are expressed as means ± standard error (SEM) and analyzed with the statistical analysis software GraphPad Prism, Version 5.0 (GraphPad, San Diego, CA, USA). All data were analyzed using one-way analysis of variance (ANOVA) except for the data from the acquisition training of the water maze, coat state and body weight, which were analyzed using two-way ANOVA. Bonferroni’s tests were used for *post hoc* multiple treatment comparisons. Statistical significance was considered when *P* < 0.05.

## Additional Information

**How to cite this article**: Wang, Z.-Z. *et al.* Phosphodiesterase-4D Knock-down in the Prefrontal Cortex Alleviates Chronic Unpredictable Stress-Induced Depressive-Like Behaviors and Memory Deficits in Mice. *Sci. Rep.* **5**, 11332; doi: 10.1038/srep11332 (2015).

## Supplementary Material

Supplementary Information

## Figures and Tables

**Table 1:** Chronic Unpredictable Stress Procedure.

Day	Stressors		
	Morning	Afternoon	Night
1	cold swimming (6 min, in 10 °C water)	sawdust-free cage (8–12 h)	overnight illumination
2	restraint stress (1 h)	damp sawdust (8–12 h )	_
3	electric footshock (0.8 mA, 5-s duration, 60-s inter-shock interval for 1 h)	45° cage tilting (8–12 h )	food deprivation (overnight)
4	tail pinch (1 cm apart from the end of the tail, 1 min)	sawdust-free cage with 200 ml water (21 °C, 8 −12 h )	_
5	overhanging (30 min)	45° cage tilting (8–12 h )	_
6	white noise (1 h)	restraint stress (1 h)	_
7	sawdust-free cage (8 -12 h)	cold swimming (6 min, in 10 °C water)	water deprivation (overnight)
8	continuous cage shaking (1 h)	sawdust-free cage with 200 ml water (21 °C, 8 −12 h )	_
9	restraint stress (1 h)	45° cage tilting (8 −12 h )	_
10	electric footshock (0.8 mA, 5-s duration, 60-s inter-shock interval for 1 h)	overhanging (30 min)	overnight illumination
11	damp sawdust (8 −12 h )	tail pinch (1 cm apart from the end of the tail, 1 min)	_
12	continuous cage shaking (1 h)	restraint stress (1 h)	food deprivation (overnight)
13	overhanging (30 min)	cold swimming (6 min, in 10 °C water)	_
14	white noise (1 h)	sawdust-free cage with 200 ml water (21 °C, 8 −12 h )	overnight illumination
