# Supplementary material for: Phosphodiesterase-4D Knock-down in the Prefrontal Cortex Alleviates Chronic Unpredictable Stress-Induced Depressive-Like Behaviors and Memory Deficits in Mice
Source: Sci Rep. 2015 Jul 10;5:11332. doi: 10.1038/srep11332 (PMC4498385; doi:10.1038/srep11332)
Supplement: Supplementary Information [file srep11332-s1.pdf]

Phosphodiesterase-4D Knockdown in the Prefrontal Cortex Alleviates Chronic Unpredictable Stress-Induced Depressive-Like Behaviors and Memory Deficits in Mice

Zhen-Zhen Wang[1,2], Wei-Xing Yang[1,3], Yi Zhang[4], Nan Zhao[1], You-Zhi Zhang[1], Yan-Qin Liu[1], Ying Xu[6], Steven P. Wilson[5], James M. O'Donnell[6], Han-Ting Zhang[7] & Yun-Feng Li[1]

[1]Department of New Drug Evaluation, Beijing Institute of Pharmacology and Toxicology, Beijing 100850, China, [2]State Key Laboratory of Bioactive Substances and Functions of Natural Medicines, Institute of Materia Medica & Neuroscience Center, Chinese Academy of Medical Sciences and Peking Union Medical College, Beijing 100050, China, [3]Department of Pharmacology, China Pharmaceutical University, Nanjing 210009, China, [4]Department of Anatomy, School of Preclinical Medicine, Beijing University of Chinese Medicine, Beijing 100029, China, [5]Department of Pharmacology, Physiology, and Neuroscience, University of South Carolina School of Medicine, Columbia, SC 29208, USA, [6]School of Pharmacy & Pharmaceutical Sciences, The State University of New York at Buffalo, New York, NY 14260, USA, [7]Departments of Behavioral Medicine & Psychiatry and Physiology & Pharmacology, West Virginia University Health Sciences Center, Morgantown, WV26506, USA

Correspondence and requests for materials should be addressed to Y. F. L. (lyf619@aliyun.com)

Supplementary Information

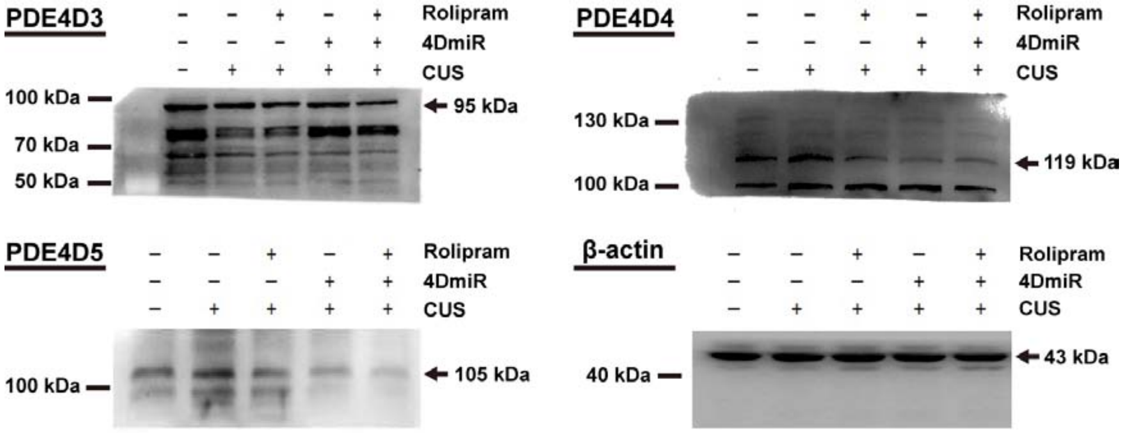

Supplementary Figure S1 | Representative blots of long-form PDE4D variants and *β*-actin in the prefrontal cortical tissues. The prefrontal cortical tissues of 3 mm in diameter around the injection site were punched out for western blot analysis. The black bars beside the images indicate the position of molecular size markers. The black arrowheads indicate the position of the target proteins shown in Fig. 1b in the main article. All gels were run under the same experimental conditions and blots were processed in parallel.

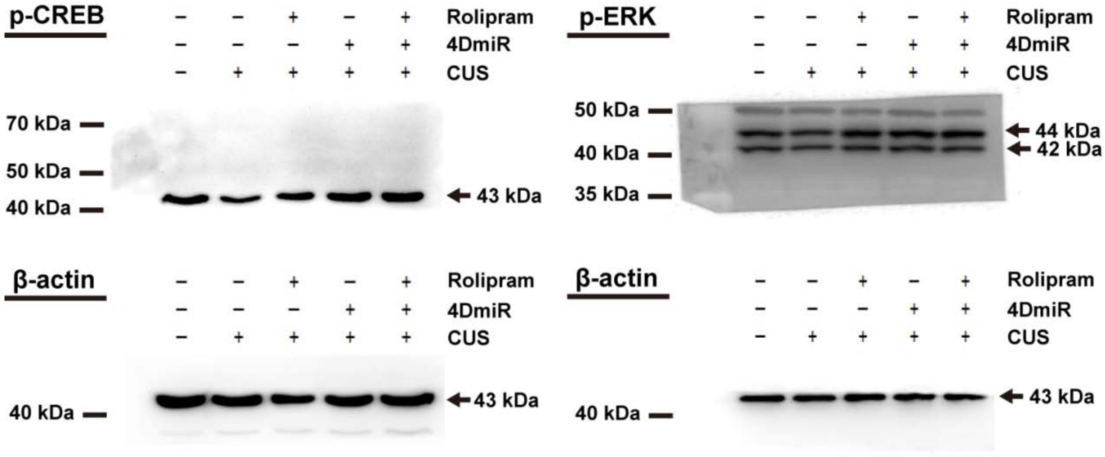

Supplementary Figure S2 | Representative blots of pCREB, pERK and *β*-actin in the prefrontal cortical tissues. The prefrontal cortical tissues of 3 mm in diameter around

the injection site were punched out for western blot analysis. The black bars beside the images indicate the position of molecular size markers. The black arrowheads indicate the position of the target proteins shown in Fig. 6c and d in the main article. All gels were run under the same experimental conditions and blots were processed in parallel.
